# Long-term survival in a patient with postoperative pulmonary oligometastasis of pancreatic cancer treated with surgical resection and chemotherapy: A case report

**DOI:** 10.1097/MD.0000000000043647

**Published:** 2025-08-01

**Authors:** Naotake Funamizu, Noriko Funamizu, Yoshinaru Hirose, Yoshiaki Kamei, Riko Kitazawa, Yuzo Umeda, Tsunemichi Hirose

**Affiliations:** a Department of Gastroenterology, Hirose Hospital, Imabari, Ehime, Japan; b Department of Hepatobiliary, Pancreatic, and Breast Surgery, Ehime University Graduate School of Medicine, Toon, Ehime, Japan; c Department of Diagnostic Pathology, Ehime University Graduate School of Medicine, Toon, Ehime, Japan.

**Keywords:** case report, chemotherapy, long-term survival, metastasectomy, pancreatic cancer, pulmonary oligometastasis

## Abstract

**Rationale:**

Pancreatic cancer (PC) frequently recurs after curative surgery. Pulmonary metastases are uncommon but may indicate more indolent disease. The benefit of aggressive treatment in cases with minimal dissemination remains unclear.

**Patient concerns:**

A 77-year-old man developed a solitary pulmonary nodule 25 months after distal pancreatectomy for stage IB PC. Tumor markers remained within normal range.

**Diagnoses:**

Thoracoscopic resection confirmed metastatic PC. Pleural lavage cytology was positive, indicating microscopic dissemination.

**Interventions:**

Systemic chemotherapy was administered for over 2 years using various regimens: gemcitabine + nab-paclitaxel, nano-liposomal irinotecan + fluorouracil + leucovorin, gemcitabine + S-1, and modified fluorouracil + leucovorin + irinotecan + oxaliplatin. Treatment was paused due to fatigue when the disease and markers stabilized.

**Outcomes:**

After treatment interruption, a recurrent pleural mass emerged with elevated CA19-9. Chemotherapy was resumed, achieving renewed control. All standard regimens were eventually exhausted, and genomic profiling revealed no actionable mutations. The patient remains alive and stable 4.7 years after metastasis, but the optimal treatment duration remains unclear.

**Lessons:**

This case illustrates the challenge of managing chemosensitive but incurable PC with oligometastasis and minimal dissemination. It highlights the limitations of tumor markers for early recurrence detection and the difficulty in determining treatment length when therapeutic options are depleted. Individualized, sustained therapy can offer prolonged disease control. In this case, systemic treatment was continued beyond standard timelines, prioritizing disease suppression despite treatment fatigue.

## 1. Introduction

Pancreatic cancer (PC) is among the most aggressive and lethal malignancies, with a 5-year overall survival rate of approximately 10% in the United States.^[1]^ Survival outcomes differ markedly by disease stage, with reported rates of 44% for localized disease, 16% for regional spread, and as low as 3% for distant metastases.^[1,2]^ Despite improvements in surgical techniques and systemic therapies, recurrence after curative-intent resection remains common, affecting up to 80% of patients.^[3]^

The predominant patterns of recurrence include local relapse, hepatic metastases, and peritoneal dissemination. In contrast, pulmonary metastases are relatively uncommon, occurring in an estimated 13.6% of cases.^[4]^ Nevertheless, emerging evidence suggests that patients with isolated pulmonary oligometastases may exhibit a more favorable prognosis than those with liver or peritoneal involvement.^[5,6]^ Several case reports have also described long-term survival following surgical resection of isolated pulmonary metastases from PC, highlighting the potential benefit of metastasectomy in highly selected patients.^[7,8]^ Furthermore, surgical resection of limited pulmonary metastases has been associated with prolonged survival in carefully selected patients.

Here, we report a rare case of a patient who developed delayed-onset pulmonary oligometastasis following distal pancreatectomy for stage IB PC. Despite cytologically confirmed pleural dissemination, the patient achieved long-term survival exceeding 4.5 years after recurrence through a combination of pulmonary metastasectomy and prolonged, individualized systemic chemotherapy. This case underscores the potential role of aggressive, multidisciplinary treatment in achieving meaningful disease control in patients with oligometastatic PC.

## 2. Case report

A 70-year-old man was diagnosed with PC and underwent distal pancreatectomy at Ehime University Hospital. Histopathological examination revealed a moderately differentiated adenocarcinoma, staged as pT2N0M0, corresponding to stage IB according to the 7th edition of the Union for International Cancer Control (UICC) classification. Following Japanese guidelines, adjuvant chemotherapy with oral S-1 was initiated and completed over 6 months (100 mg/day, 4-week on/2-week off cycles for 4 courses).^[9]^

**Figure 1.**
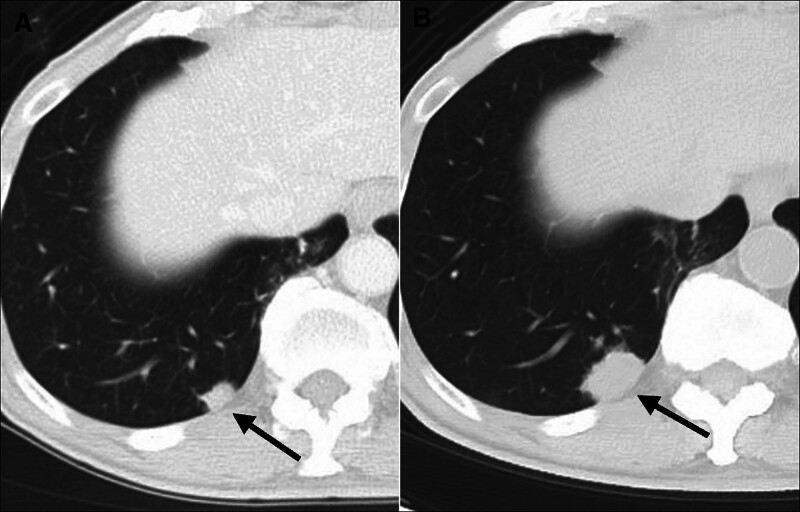
Radiological progression of pulmonary oligometastatic lesion following pancreatectomy. (A) Chest computed tomography (CT) at 25 months post-pancreatectomy revealed a 10-mm solitary solid nodule in the right lower lobe (black arrow), suggestive of pulmonary metastasis or primary lung cancer. (B) A follow-up CT 2 months later showed progressive lesion growth to 20 mm in diameter, indicating the need for surgical intervention.

Postoperative surveillance imaging with computed tomography (CT) revealed no recurrence at 21 months. However, at 25 months postoperatively, a solitary 10-mm nodule was detected in the right lower lobe of the lung (Fig. 1A). Although tumor markers remained within normal limits, the lesion gradually increased to 20 mm over 2 months (Fig. 1B). Given the suspicion of either primary lung cancer or pulmonary metastasis from PC, a CT-guided needle biopsy was attempted but yielded inconclusive results. To obtain a definitive diagnosis and for therapeutic intent, thoracoscopic partial right lower lobe resection was performed at 31 months post-pancreatectomy.

**Figure 2.**
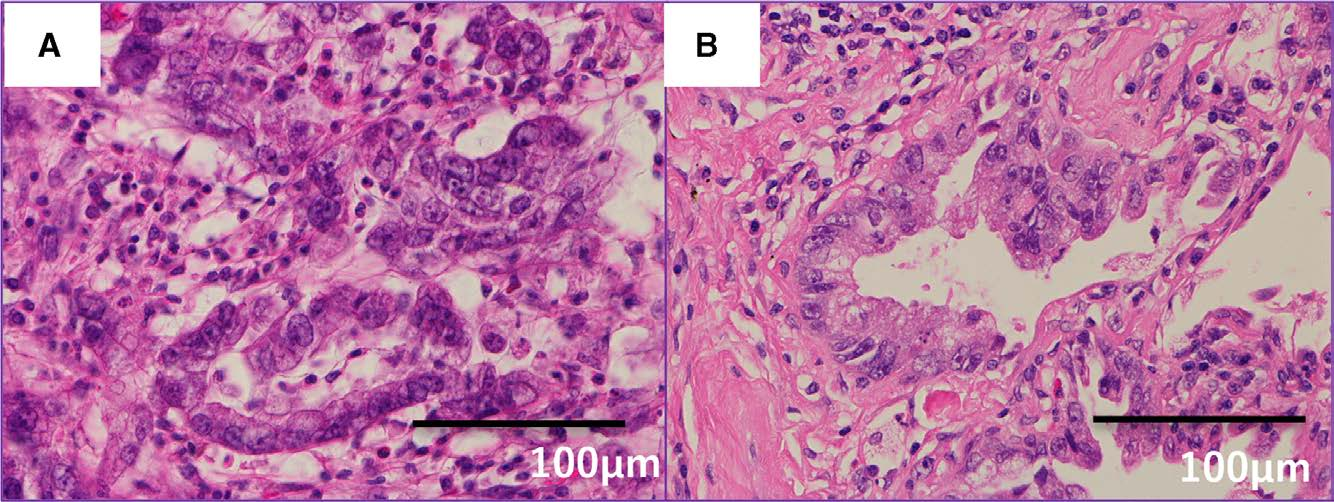
Histopathological comparison between primary pancreatic tumor and pulmonary metastasis. (A) Hematoxylin and eosin staining of the primary pancreatic tumor showed moderately differentiated adenocarcinoma (×400). (B) The resected pulmonary lesion showed histological features consistent with moderately differentiated adenocarcinoma, similar to the primary pancreatic tumor, supporting the possibility of pulmonary metastasis from pancreatic cancer (H&E, ×400).

**Figure 3.**
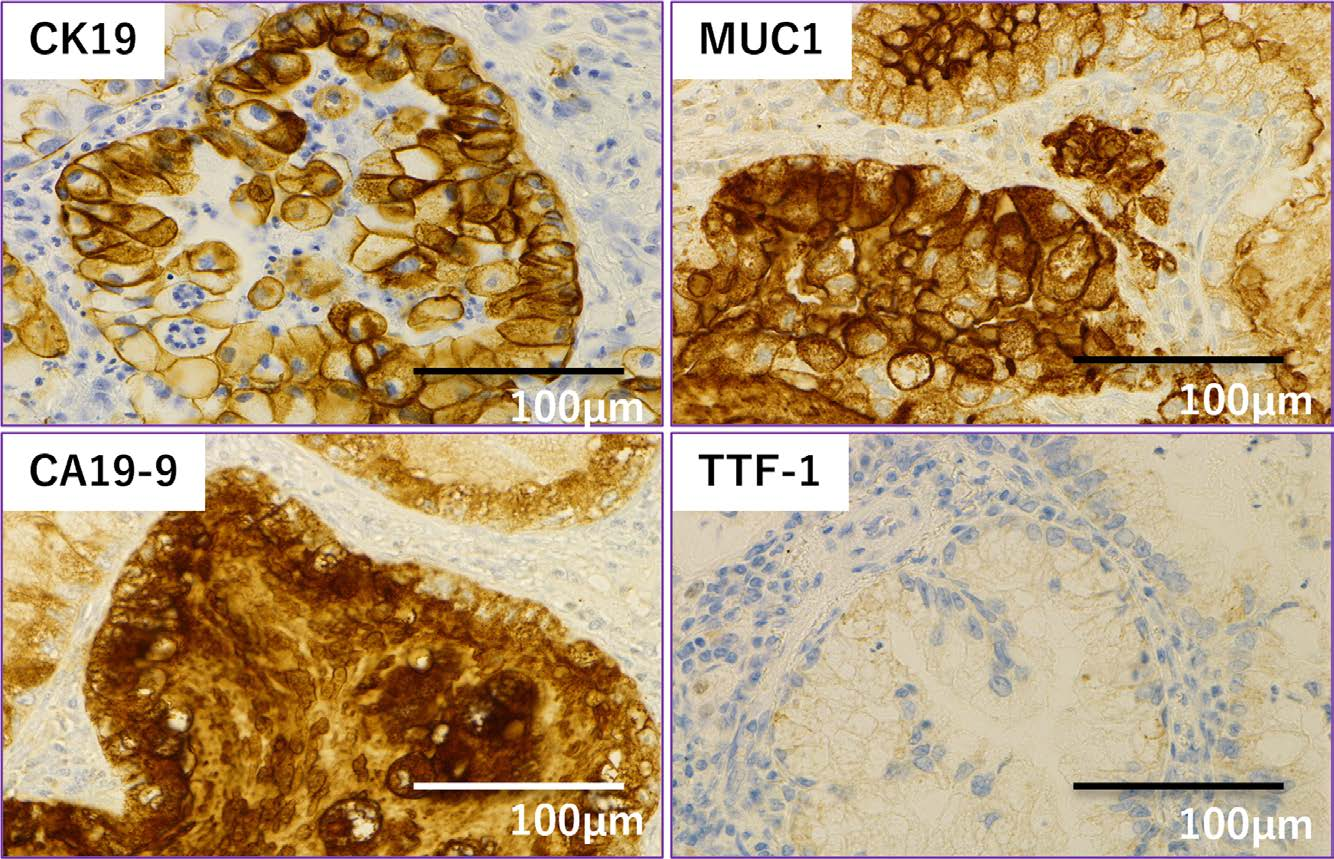
Immunohistochemical staining of pulmonary metastasis from pancreatic cancer. This figure shows the immunohistochemical profiles of a metastatic lung lesion originating from pancreatic carcinoma. The tumor cells are strongly positive for CK19, MUC1, and CA19-9, consistent with the immunophenotype of pancreatic origin. In contrast, thyroid transcription factor-1 (TTF-1), a marker typically expressed in primary lung adenocarcinomas, is negative. These findings support the diagnosis of pulmonary metastasis from pancreatic carcinoma rather than a primary lung tumor (×400).

Hematoxylin and eosin (H&E) staining revealed similar histological features between the primary pancreatic tumor (A) and the pulmonary lesion (B), both showing moderately differentiated adenocarcinoma (Fig. 2A and B). Histological analysis revealed features consistent with the primary pancreatic tumor, and cytological examination of pleural lavage fluid was positive for malignant cells. Immunohistochemical staining was negative for thyroid transcription factor-1, and positive for CK19, MUC1, and CA19-9 (Fig. 3), confirming pulmonary metastasis from PC.

Following surgery, chemotherapy with gemcitabine plus nab-paclitaxel was initiated. The doses were calculated based on the patient’s body surface area (1.52 m²): gemcitabine 850 mg/m² and nab-paclitaxel 100 mg/m², administered in a 3-week-on/1-week-off cycle. However, the regimen was discontinued after 3 cycles due to generalized fatigue.

Subsequently, the treatment regimen was changed to nano-liposomal irinotecan plus 5-fluorouracil and leucovorin (nal-IRI + 5-FU/LV), comprising nal-IRI 100 mg, 5-FU 3200 mg (46-hour continuous infusion), and L-leucovorin 350 mg/m² biweekly. Genetic testing for UGT1A1 (*6 heterozygous) revealed low risk for severe adverse events. The patient tolerated 12 cycles of this regimen without major toxicity.

Comprehensive genomic profiling, including microsatellite instability testing, revealed no actionable mutations. The patient subsequently requested a break from chemotherapy. As imaging showed no new lesions and tumor markers remained stable, treatment was temporarily discontinued. S-1 monotherapy (100 mg/day) was administered as maintenance therapy for 6 months, with monthly tumor marker surveillance and contrast-enhanced CT every 3 months.

**Figure 4.**
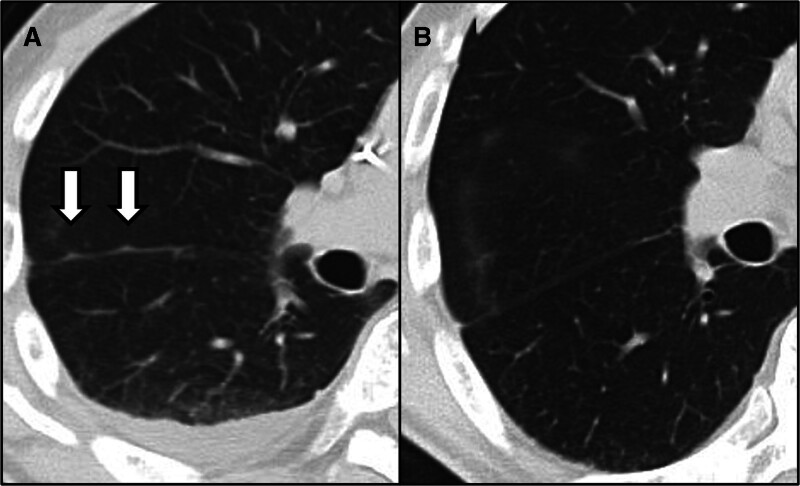
Radiologic findings of pleural dissemination and response to chemotherapy. (A) Chest CT performed at recurrence revealed multiple small nodular opacities along the interlobar pleura, raising suspicion of pleural dissemination. (B) CT imaging obtained 5 months after chemotherapy re-initiation showed complete radiological disappearance of the nodular lesions.

At 51 months after initial surgery and 26 months after lung metastasectomy, an increase in tumor markers and pleural dissemination was detected (Fig. 4A). Chemotherapy was resumed using the nal-IRI + 5-FU/LV regimen. Serum CA19-9 levels, measured by electrochemiluminescence immunoassay, gradually decreased and returned to within the normal range (<37 U/mL) after 5 months of chemotherapy. Concurrently, follow-up imaging showed disappearance of the disseminated lesions (Fig. 4B). However, due to fatigue, treatment was again discontinued.

Gemcitabine monotherapy (1000 mg/m², 3-weeks-on/1-week-off) was initiated but was later switched to gemcitabine + S-1 therapy (gemcitabine 1000 mg/m² and oral S-1 at 80 mg/day for 2 weeks every 3 weeks) due to rising CA19-9 levels. After 2 months, CA19-9 continued to increase, prompting a transition to modified FOLFIRINOX (5-FU 3200 mg continuous infusion, leucovorin 300 mg, irinotecan 220 mg, oxaliplatin 120 mg) biweekly.

**Figure 5.**
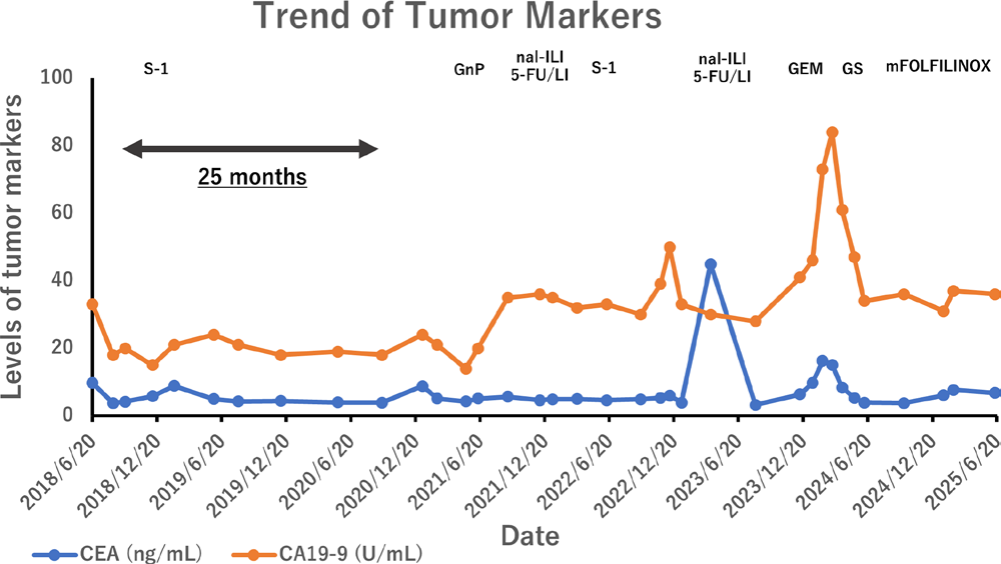
Longitudinal trend of tumor markers throughout the clinical course. Serial measurements of carcinoembryonic antigen (CEA, blue line) and carbohydrate antigen 19-9 (CA19-9, orange line) were tracked from initial surgery (June 2018) through the latest follow-up (April 2025). Arrows indicate key clinical interventions, including surgical resection and initiation of chemotherapy. Treatment regimens are indicated by horizontal bars along the top axis, representing the duration of each chemotherapy protocol. Peaks in CA19-9 correspond to disease recurrence, while subsequent decreases reflect response to therapy. CEA levels remained relatively stable throughout, with 1 transient elevation.

Following 8 cycles of FOLFIRINOX, serum CA19-9 level decreased to within the normal range (<37 U/mL), indicating a favorable biochemical response. However, the patient developed peripheral neuropathy. To manage this toxicity, the treatment interval was first extended to every 3 weeks, and subsequently to every 4 weeks, during which stable disease was confirmed on follow-up imaging. After an additional 4 months of treatment, oxaliplatin was removed from the FOLFIRINOX regimen due to worsening neuropathy. The modified chemotherapy, without oxaliplatin, was continued for 3 more months, during which both stable disease and stable CA19-9 levels were maintained (Fig. 5).

As of April 2025, the patient remains alive and on chemotherapy, 4 years and 7 months after recurrence of pulmonary oligometastasis, and 6 years and 8 months after initial surgery. No new lesions have been identified to date.

## 3. Discussion

This case highlights several clinical challenges in managing recurrent PC, particularly in patients with isolated pulmonary oligometastasis and no actionable genomic alterations. Although adjuvant S-1 chemotherapy was completed without complication, recurrence still occurred, underscoring the need for long-term surveillance even after apparent therapeutic success.

We have previously demonstrated that the C-reactive protein-to-albumin ratio is a useful predictor of tolerability to S-1 chemotherapy,^[10]^ and this finding has been externally validated.^[11]^ Additionally, we identified the geriatric nutritional risk index as another independent marker for predicting S-1 tolerability.^[12,13]^ While these markers are practical tools for tailoring adjuvant treatment based on our clinical data, they do not predict recurrence itself. Therefore, continued surveillance is necessary, even in patients who complete therapy successfully.

Pulmonary metastases from PC are considered a biologically distinct subset and may show a more indolent clinical course in some patients.^[14,15]^ In this case, recurrence appeared more than 2 years postoperatively with normal tumor markers, suggesting a biochemically and radiographically silent progression.

The pathophysiology of isolated lung metastasis is not fully understood. While Weiss Cascade theory emphasizes liver-predominant spread via the portal system,^[16]^ alternative hematogenous or lymphatic dissemination, particularly after distal pancreatectomy, may explain isolated pulmonary recurrence.^[17]^

Surgical resection of pulmonary oligometastases has demonstrated survival benefits in selected patients.^[18–20]^ Japanese reports have shown favorable outcomes even when metastasectomy is performed several years after the primary surgery.^[21–23]^ In this case, no pleural effusion was observed preoperatively, and thoracoscopic resection was considered appropriate and carried out as planned. During the procedure, malignant cells were unexpectedly detected via pleural lavage cytology. Nevertheless, in light of primary tumor control, limited disease extent, and resectability, as outlined by the Thomford criteria,^[22]^ the decision to proceed with surgery was deemed justified.

After resection, the patient received sequential chemotherapy including gemcitabine plus nab-paclitaxel, nal-IRI + 5-FU/LV, GS, and modified FOLFIRINOX. Each treatment interruption due to fatigue was followed by disease progression, whereas resumption of therapy resulted in disease control. This underscores the value of sustained systemic therapy tailored to patient tolerability and tumor responsiveness.

Importantly, tumor marker profiles evolved during the course. Initial pulmonary recurrence was marker-negative, but CA19-9 and CEA rose significantly during progression. This phenotypic shift reflects clonal evolution under therapeutic pressure and highlights the need for multimodal monitoring, including imaging and cytology.

Comprehensive genomic profiling failed to reveal actionable mutations. Such scenarios are common in advanced PC, and emphasize the ongoing importance of optimizing conventional cytotoxic chemotherapy.

Although surge ry is not routinely recommended for recurrent PC, selected cases may benefit from reoperation when the disease is localized and controlled.^[24]^ This case aligns with the oligometastasis model proposed by Hellman and Weichselbaum,^[25]^ whereaggressive local and systemic therapies can achieve long-term disease control. Despite pleural cytology positivity and exhaustion of standard regimens, the patient remains alive nearly 5 years after recurrence and over 6 years after initial surgery, demonstrating the potential impact of sustained, individualized multidisciplinary care.

## 4. Conclusion

This case illustrates that long-term survival is achievable in recurrent PC with isolated pulmonary oligometastasis through a combination of metastasectomy and sustained, individualized chemotherapy. Even in the presence of pleural lavage cytology positivity and the absence of actionable mutations, careful patient selection, close monitoring, and flexible adaptation of standard therapies can offer meaningful disease control. This experience supports the application of the oligometastasis concept in PC and highlights the clinical value of persistence in treatment beyond conventional expectations.

## Acknowledgments

The authors would like to thank Enago (www.enago.jp) for the English language review.

## Author contributions

**Data curation:** Naotake Funamizu, Noriko Funamizu.

**Investigation:** Yoshiaki Kamei.

**Project administration:** Naotake Funamizu, Riko Kitazawa, Yuzo Umeda.

**Writing – original draft:** Naotake Funamizu.

**Writing – review & editing:** Noriko Funamizu, Yoshinaru Hirose, Yoshiaki Kamei, Riko Kitazawa, Yuzo Umeda, Tsunemichi Hirose.
